# Can modeling of HIV treatment processes improve outcomes? Capitalizing on an operations research approach to the global pandemic

**DOI:** 10.1186/1472-6963-8-166

**Published:** 2008-08-04

**Authors:** Wei Xiong, Nathaniel Hupert, Eric B Hollingsworth, Megan E O'Brien, Jessica Fast, William R Rodriguez

**Affiliations:** 1 Department of Public Health, Weill Medical College, Cornell University, New York, NY, USA; 2 Department of Medicine, Weill Medical College, Cornell University, New York, NY, USA; 3 NewYork-Presbyterian Hopital, New York, NY, USA; 4 Clinton Foundation HIV/AIDS Initiative (CHAI), Quincy, MA, USA; 5 Global Health Delivery Project, Brigham and Women's Hospital, Harvard Medical School, Boston, MA, USA; 6 Partners AIDS Research Center, Massachusetts General Hospital, Boston, MA, USA

## Abstract

**Background:**

Mathematical modeling has been applied to a range of policy-level decisions on resource allocation for HIV care and treatment. We describe the application of classic operations research (OR) techniques to address logistical and resource management challenges in HIV treatment scale-up activities in resource-limited countries.

**Methods:**

We review and categorize several of the major logistical and operational problems encountered over the last decade in the global scale-up of HIV care and antiretroviral treatment for people with AIDS. While there are unique features of HIV care and treatment that pose significant challenges to effective modeling and service improvement, we identify several analogous OR-based solutions that have been developed in the service, industrial, and health sectors.

**Results:**

HIV treatment scale-up includes many processes that are amenable to mathematical and simulation modeling, including forecasting future demand for services; locating and sizing facilities for maximal efficiency; and determining optimal staffing levels at clinical centers. Optimization of clinical and logistical processes through modeling may improve outcomes, but successful OR-based interventions will require contextualization of response strategies, including appreciation of both existing health care systems and limitations in local health workforces.

**Conclusion:**

The modeling techniques developed in the engineering field of operations research have wide potential application to the variety of logistical problems encountered in HIV treatment scale-up in resource-limited settings. Increasing the number of cross-disciplinary collaborations between engineering and public health will help speed the appropriate development and application of these tools.

## 1. Background

Three decades after its start, the global HIV epidemic is now the target of perhaps the most ambitious global health program ever conceived, dwarfing in its complexity and requirement for sustainability the campaign to eradicate smallpox, which is widely considered one of the crowning public health achievements of the late 20^th ^century. In the last ten years, the campaign to scale up HIV care and antiretroviral therapy (ART) for millions of people with AIDS living in resource-limited countries has gained substantial commitments in financing [e.g., the Global Fund to Fight AIDS, Tuberculosis and Malaria (GFATM), the World Bank's Multi-Country AIDS Program (MAP), the United States' President's Emergency Plan for AIDS Relief (PEPFAR) program, private donors such as the Bill & Melinda Gates Foundation], operational support [e.g., Joint United Nations Programme on HIV (UNAIDS), World Health Organization (WHO)], and procurement support [e.g., the Clinton Foundation HIV/AIDS Initiative (CHAI)].

While over 30 million people are living with HIV and over 2 million new infections are estimated to occur each year, HIV care and treatment has expanded significantly in this decade: it is estimated that by December 2006 more than 2 million people, or 28% of people living with HIV in low- and middle-income countries, had access to antiretroviral treatment, a substantial improvement from 2% coverage just three years earlier [1-3]. These figures confirm the success of early efforts to scale up global HIV treatment, but serious obstacles to continued expansion remain to be addressed. For example, approximately 95% of HIV-infected people live in developing countries [4], which typically face challenges of underfunding, limited materiel resources, and severe human resource shortages in the health sector [5]. Increasing ART coverage in these settings will require sophisticated logistics and managerial skill at every point of health service delivery from the local to the transnational levels. The operational challenges include developing adequate and efficient physical infrastructures, providing long-term financial sustainability, and eliminating constraints to treatment capacity, most notably in human resources and pharmaceuticals and diagnostics supply chains.

These challenges were noted in a recent review of the PEPFAR program: "The continuing challenge for the U.S. Global AIDS Initiative is to simultaneously maintain the urgency and intensity that have allowed it to support a substantial expansion of HIV/AIDS services in a relatively short time while also placing greater emphasis on long-term strategic planning and increasing the attention and resources directed to capacity building for sustainability [5]." Below we describe one scientific field that holds promise to help maintain the acceleration of HIV treatment scale-up: the discipline of Operations Research (OR), the applied science of maximizing the effective use of limited resources [6].

OR offers a plethora of modeling techniques that may be used to assess, operationalize, and evaluate HIV treatment scale-up activities with the goal of transforming existing health systems in target countries to achieve efficiency and sustainability in the provision of life-long, effective treatment for people with HIV. While OR techniques and models have been successfully utilized in many areas to maximize the effective use of scarce resources, the application of mathematical modeling to HIV scale-up to date typically has focused on policy-level issues (e.g., program costs) as opposed to operational-level issues [7-10]. We believe that application of these tools to practical decisions about resource allocation and health care organization may increase the success of existing programs and help to expand access to HIV care and treatment in resource-limited countries. This paper reviews current challenges to expanded HIV care and treatment that may be amenable to OR-based interventions; discusses potential benefits and pitfalls of the use of applied models in longitudinal HIV care and treatment; and concludes with a summary to guide the future application of OR to scale-up activities.

## 2. The practical challenges of HIV treatment scale-up

Global scale-up of HIV care and treatment involves complex issues in logistics, the science that deals with the procurement, distribution, maintenance, and replacement of materiel and personnel [11]. During the WHO's "3 by 5" campaign, many stakeholders provided logistical support, including procurement and distribution of antiretroviral drugs and other supplies, creation of infrastructure, and the training and recruitment of the healthcare work force. Not surprisingly, significant logistical challenges remain. Here we focus on three categories of logistical challenges that are key barriers to achieving universal access to HIV prevention and treatment: pharmaceutical supply chain management, laboratory service infrastructure and planning, and healthcare workforce development.

### 2.1 Pharmaceutical supply chain management

The increasing demand of global AIDS treatment poses unprecedented challenges for supply chain management, since each point of dispensing antiretroviral (ARV) drugs (i.e., clinic, hospital, community outreach worker) must have access to a customized and predictable supply of ARVs and other drugs at all times. The robustness of this supply chain is essential to the success of any AIDS treatment program, and it must take into account a number of unique features and constraints of antiretroviral treatment.

At the clinical level, HIV treatment scale-up in most low- and middle-income countries embraces the WHO's "public health approach" to ART use, where treatment regimens are standardized according to widely accepted consensus guidelines [12]. Unavoidably, customization of care takes place at multiple points, from country-level protocols that adapted WHO guideline to regional needs, to patient-level adjustments in treatment due to co-morbidities, such as tuberculosis, or adverse events, such as stavudine-related neuropathy. The degree of customization varies considerably from program to program; in Brazil, for example, clinicians have access to most antiretroviral medications, while in Malawi individual health facilities receive shipments of a prescribed, narrow formulary [13,14]. In all settings, key patient-level variables (e.g., the presence of anemia or neuropathy) should trigger a range of possible drug substitutions that will lead to measurable differences in demand at an individual clinic or regional level of care. The ideal pharmaceutical supply chain that is responsive to these needs will have, at a minimum, the following features [15]:

(1) an inventory control system

(2) a logistics management information system

(3) a storage system, and

(4) a distribution system

Major efforts are underway to assist countries and care providers with all four of these aspects of supply chain development and management. To be successful, these efforts need to overcome a number of constraints, some of which are unique to ART and some of which apply generally to medical material management. ARVs are notable for their short shelf-lives and, for some – particularly the non-heat stable form of protease inhibitors – the need for a "cold chain" to provide refrigeration. Standard ART formularies for children and adults require multi-drug regimens that often involve products manufactured by more than one manufacturer, potentially complicating efforts to insure not only continuity but parity of supplies. For many other medical conditions, including many infectious diseases, drug substitution due to stockouts (e.g., prescribing one antibiotic for another that is not available) is common and has little clinical impact. Stockouts of ARVs, in contrast, can have important health consequences because of the limited availability and medical compatibility of substitute drugs, and the serious risks of intermittent adherence in terms of viral resistance. Finally, although the cost of first-line ARVs has declined dramatically in recent years, ART is one of the most expensive global public health interventions ever undertaken, amplifying the impact of supply chain inefficiencies [16].

The ideal supply chain will synchronize aggregate patient-level drug demand with the flow of pharmaceuticals from suppliers, reducing inventory investment along the chain, and improving patient care by minimizing supply shortages. Sustainable and flexible supply chains with capacity for forecasting, procurement, distribution, and information sharing can improve drug utilization and decrease waste, yet even the best-designed supply chains may experience interruptions caused by shortage, damage, drug expiration, and miscommunication. Optimizing supply chain management (SCM) has been a central focus of the OR community since the concept of SCM first appeared in the engineering literature in the mid-1980's, bridging previously independent lines of research into inventory control, product management, and industrial processes control [17,18].

### 2.2 Laboratory service infrastructure and planning

Laboratory-based diagnostic services are necessary for the proper identification of HIV disease and are used for longitudinal monitoring of all HIV-infected patients. Laboratory data – notably the CD4 cell count – can guide the timing of therapeutic interventions and ensure the maximum level of safety and monitoring for treatment failure when antiretroviral drugs are being delivered [1]. However, access to hospital-based laboratory facilities for ART monitoring is a challenge in most resource-limited countries; to compound matters, outpatient laboratory services especially are scarce in rural areas [19]. Despite the importance of laboratory capacity for the effective delivery of ART, there have been only a handful of systematic approaches developed and implemented in resource-limited countries to ensure sustainable, practical laboratory service for HIV treatment scale-up [20].

As with the creation of effective ARV supply chains, the development and support of clinical laboratory infrastructure to support ART scale-up in resource-limited countries will need to address a number of logistical constraints. Foremost among these is the expense of currently available HIV laboratory testing equipment and reagents, their need for regular maintenance, and the skilled labor force required both to operate and maintain them. Currently, many countries operate a decentralized model of laboratory capacity with little interaction between laboratories at national, regional, and district levels [20]. In some of these countries, many facilities dispensing ART already experience shortages of functioning equipment, reagents, and technicians to conduct regular maintenance and technical support. When faced with insufficient laboratory capacity, health planners have two options: add additional laboratory capacity by purchasing more equipment, or increase the utilization of equipments that have already been purchased and are either idle or underutilized, often through the implementation of a sample transport network. Ancillary costs, such as those of reagents, typically are tied in complex ways to machine utilization, so the cost of an idle machine may be more than simply its inconvenience or the downstream costs of lost clinical services.

After ARV costs, building laboratory capacity is the most capital-intensive component of global ART scale-up. In addition to technical training and patient education, it involves equipment selection, human resource training for operations and maintenance, reagent selection and purchase, and transportation infrastructure for moving samples and results between points of care and laboratory machines [21]. Less well described is the need for laboratory information technology infrastructure, to link samples and results to patients, and return the laboratory data from the testing facilities to the clinicians at the sites of care. It is possible that, with proper development of infrastructure for training, preventive maintenance, and repairs, many countries currently experiencing laboratory service interruptions have sufficient laboratory capacity for ART scale-up. If this is the case, in the short term, rather than adding more machines, Ministries of Health may be better served by development of a flexible and efficient sample referral network to bring samples to the instruments which are currently under-utilized, thereby ensuring the direct and indirect benefits from using that laboratory capacity at its maximum potential. In the long term, investments in laboratory infrastructure and the development of more appropriate, point-of-care devices will be needed to replace potentially unreliable specimen transport networks and shorten the cycle time of laboratory-based information back to the clinical encounter, which can be as long as several weeks in many settings.

### 2.3 Healthcare workforce development

In addition to supply chain management and laboratory capacity, the challenge of training and retention of healthcare workers is a third critical logistical aspect of HIV service delivery that has been widely identified as perhaps the single largest constraint on global treatment scale-up. This challenge is greatest in regions worst-hit by the disease due to multiple factors including direct workforce effects of the pandemic (i.e., death and disability), international financing policies restricting investment in the health sector, and the "brain drain" – outmigration of qualified practitioners toward urban areas and higher wage countries in Europe and North America [22]. Human resources affect both the scope and speed of treatment scale-up in most resource-limited countries, yet few countries have a "comprehensive training plan, a clear assessment of ongoing training needs, a plan to operationalize training on a large scale, or adequate funds budgeted for training [23]."

The spectrum of healthcare workers encompasses doctors, nurses, pharmacists, lab technicians, phlebotomists, counselors, program managers, and community health workers, as well as other ancillary staff. Successful scale-up involves relatively complex planning for recruiting and training a mix of healthcare workers providing a wide range of services, which are influenced significantly by the HIV care delivery model chosen by a country or region. In a setting where the demand for healthcare workers enormously exceeds the available resources, OR approaches may be able to provide technical support to program mangers to evaluate the capacity of existing systems and estimate the minimal required amount of healthcare workers for effective scale-up. Furthermore, OR models can be used to evaluate different models of care and to provide insight into the impact on quality and capacity of HIV clinics adopting different HIV care delivery models.

## 3. Applying Operations Research to Scale-up

Millions of people living in resource-limited countries need life-long medical care, now including ART, posing a fundamental logistical challenge for effective HIV care and treatment and raising challenges to the technical feasibility of the goal of universal access. Increased funding alone, though necessary, will not solve these issues, since it is not only the procurement of resources but the management of those resources at the point of need that will determine the success or failure of local HIV interventions. This means there is an urgent need for strategies to build and maintain efficient systems of care and pharmaceutical delivery, laboratory capacity, and healthcare worker recruitment and training. In the remainder of this paper, we show how these areas may benefit from the application of operations research methods, as well as anticipated barriers to this application.

### 3.1 What is Operations Research?

In the world of HIV treatment and prevention, the term "operations research" or, more commonly, "operational research" has been applied to a field of study that is somewhat different from what engineers and management scientists mean by OR. To date, most HIV-related operations research studies have focused on the description, analysis, and improvement of day-to-day activities or "operations" of HIV program [24]. These operations include training, education, counseling and testing, commodity logistics, hospital and clinic activities, and community- and home-based care, among others. HIV-focused operations research therefore has been used to describe most studies that *quantify *some aspect of HIV clinic *operations *and their associated impact on patient *outcomes*. Because of this inclusive meaning, most observational field studies of ART clinic operations, for example, would be considered HIV-related operations research.

By contrast, the engineering/management science discipline of OR refers to the application of a collection of mathematical techniques used to model real-world systems and gain insight into their operations. The Institute for Operations Research and the Management Sciences (INFORMS) – the largest professional society for specialists in the field of operations research – defines operations research as "the discipline of applying advanced analytical methods to help make better decisions." This umbrella concept of operational research covers many analytic approaches and methods, such as mathematical programming, queueing theory, simulation modeling, decision analysis, and forecasting methods. Techniques from other fields such as statistics and computer science are also employed by operations researchers to assist with decision making.

OR approaches have been widely implemented for analyzing problems in complex real-life systems. These methods have provided informative insights and yielded substantial economical benefit [25], suggesting that OR models can be used in the following (sometimes overlapping) areas:

• The optimal allocation of scarce resources subject to a large number of constraints (e.g., HIV funding allocation to treatment and prevention programs)

• The search for efficient solutions among a vast multitude of alternative choices (e.g., clinical and laboratory infrastructure and capacity building)

• The analysis of dynamic systems characterized by fluctuating inputs and out puts (e.g., care delivery model evaluation and clinical activities analysis)

• The use of inferential processes to derive insights from multivariate statistical analyses (e.g., quality assurance across multiple care delivery and supply chain systems)

• Computer simulation of intricate economic and physical systems

The logistical problems arising in HIV treatment scale-up are precisely the types of problems that OR methodologies were designed to address. The challenge of scaling up HIV treatment in the developing world is unprecedented, and as such will certainly require novel solutions and new ways of thinking. However, certain core aspects of logistical problems in scale-up have been addressed successfully in other industries and settings through the use of OR methodologies.

### 3.2 How can operations research improve global HIV outcomes?

Decision makers may employ OR models to address the range of uncertainties in HIV interventions, from assessing treatment capacity requirements and the uncertainty in demand for ARVs, to the optimal staffing of treatment clinics. We categorize these logistics-related uses of OR into two types according to the scope and planning horizon considered: policy-level uses and operational-level uses.

#### 3.2.1 Policy level decision support

Policy level decisions often involve the acquisition or allocation of durable resources intended to be utilized over a long time horizon. For example, decisions related to program funding or resource allocation, healthcare workforce planning, prevention strategy or delivery model selection, typically comprise strategic-level policy decisions, and these present a clear role for the application of operations research methodologies.

##### • Resource Allocation

Mathematical programming models have been developed to address efficiency and equity in the HIV funding allocation process on the national, state, or municipal levels in the United States [8,10,26,27]. These models seek to determine the best allocation that minimizes the number of potential infections for a given period of time, given an available budget and equity considerations. Recent assessments of ART in developing countries have focused on modeling macro-level distribution of resources for health care delivery. For example, one study examined the impact of healthcare facilities location, catchment area size, and other demographic parameters on an optimally equitable allocation [27]. However, real-world HIV resource allocation typically is driven by a number of criteria aside from cost-effectiveness and equity considerations. OR-based analysis and modeling represent an evaluation methodology with which multiple competing alternatives can be weighed from multiple perspectives, including those of equity or efficiency.

##### • Healthcare Workforce Planning

Perhaps the most glaring deficiency in resource-limited settings is the lack of healthcare workers for the 30+ million people living with HIV. A variety of OR techniques are available to address problems in human resource planning, hiring, and training in knowledge-intensive operations under uncertainty [28-30]. Based on data collected on specific tasks in the delivery of care through process analysis and time-motion studies, OR models can be used to identify the optimal mix of different cadres of healthcare workers for efficient scale-up. Once targets are set for scale-up, models can also be used to estimate the demand for human resources linked to local care delivery models. More important, these estimates will contribute to understanding the capability of existing systems and identify the ideal combination of training methodologies to prepare for scale-up [23].

##### • Laboratory Infrastructure Planning

The healthcare system infrastructure in most of sub-Saharan Africa has been suffering from decades of underfunding and declining capacity [31]. Adequate financing is a fundamental requirement for building laboratory infrastructure. Yet the development of a national laboratory network into one that improves the utilization of existing laboratory capacity may result in substantial financial savings. This type of problem can be formulated as the traditional capacitated facility location problem (CFLP), which has been studied extensively in the OR literature [25]. CFLP has numerous applications in warehouse location and distribution planning, telecommunication network design, and manufacturing production planning [32]. Given accessible laboratory facilities and their processing capacity, ministries of health can use models to identify the most cost-effective way to re-distribute test demand among laboratory facilities and decide where to locate new laboratory equipment for future scale-up.

#### 3.2.2 Operational level decision support

Operational level decisions are *local *decisions related to situations where operations must be undertaken or executed in a short time frame in response to system status or changes (e.g. order quantity, equipment breakdown, weather or road changes). For instance, material managers have a difficult time determining how much safety stock to hold and when to initiate orders for material from upstream sites, given the various degrees of uncertainty in supply and demand that impact their operations. Model-based support systems can help to anticipate variability and optimize purchasing.

##### • Demand Forecasting

Forecasting is the process of estimating how much of a selected product will be needed in a given period of time. A number of tools have been developed to assist ministries of health and transnational groups in forecasting resource demand (e.g., Bertozzi, et al. [6]). However, forecasts are generally made using historical data; available data are limited since most countries started to implement their standard guidelines and protocols only in the last several years. For example, simple linear regression models have been used for global ARV demand forecasts, though they have been based on very limited observations [33]. Furthermore, commodity demands for HIV treatment programs can be highly unpredictable, because of the dynamic nature of the problem: evolving treatment protocols and patient populations requiring longitudinal follow-up with changing drug demand patterns. To improve the forecasts, attempts have been made to use a simulation approach to obtain insight into the dynamics of disease progression among existing and expected patient population, and thereby identify trends of resource usage, provide forecasts of drug demands, or even estimate the uncertainty in a forecast [34].

##### • Supply Chain Design

A supply chain is a network of facilities that performs the functions of procurement of material, transformation of material to intermediate and finished products, and distribution of finished products to customers [35]. A major challenge in managing an efficient supply chain is to minimize, to an appropriate extent, inventories and costs along the chain while maximizing customer service performance. OR has a long history of providing supply chain managers the decision support they need to design and improve a supply chain. Many industries have gained tremendous benefit from OR efforts to improve supply chain efficiency. For example, the potential savings from reengineering supply chain design ranges from $14 billion for the food service industry to $30 billion for the grocery industry [36]. However, the design and implementation of an effective HIV drug supply chain poses further challenges for both researchers and practitioners.

The strong emphasis of recent treatment scale-up efforts is to place management of pharmaceutical procurement and distribution within the primary healthcare system [5]. Treatment of HIV alone usually requires the simultaneous high-level availability (95%) of at least three drugs, each with relatively short shelf-life. In addition, side-effects of ARVs, especially in the early phase of treatment, occur with calculable but varying frequency, leading to shifting requirements for alternative first-line medications throughout the treatment coverage area. Importantly, HIV is a chronic illness associated with specific conditions that also require treatment, such as tuberculosis or cervical cancer, as well as with routine longitudinal care in a primary health setting. A recent report describes the adaptation of tuberculosis medication procurement systems with large (e.g., three-month) 'buffer stocks" in order to insure consistent availability of HIV medications, but such systems may not remain financially or logistically viable as treatment volumes increase. The unique characteristics of HIV treatment – requiring an increasing and uninterrupted supply of multiple medications in changing drug combinations – are amenable to models developed specifically for the integration of local, regional, and country-level supply chain management.

##### • Service Benchmarking

When applying the same pubic health strategies for HIV treatment programs, outcomes in terms of logistic issues will not necessarily be the same because of diversified settings across the globe. For example, the amount of inventory kept at various locations of the supply chain is an important measure of performance. However, inventories at various points may have different cost structures, varying as a result of service level requirements and supply responsiveness. It is often inappropriate to use the amounts of inventories as a direct performance metric for different supply chains and different points in a supply chain. Furthermore, the uncertainty of supply chains in different countries and regions may differ dramatically in terms of their structures, drug supplies, security, transportation and other infrastructures, etc. Therefore, in the process of scale-up, it is inappropriate to directly adopt successful inventory control rules from other programs. In order to have a better understanding and assessment of existing logistic activities, it is desirable to use OR models to determine the optimal performance needed to support a service target at various locations given the nature of the supply chain.

##### • Service Integration

The most cogent critique of many global health programs has been that their implementation involves the delivery of services in vertical silos that by themselves may improve narrowly defined outcomes, but that as a whole enable a fractured system of health care, and perpetuate both inefficiencies and inequities in health care delivery. One oft recognized aspect of the global effort to deliver HIV services is that, because HIV is inherently a longitudinal health condition best treated at a community primary care level, the effective scale-up of HIV care will necessarily target not only the disease but also prevailing vertical systems of care, addressing their inefficiencies and inequities. OR has been used in other industries to recognize the misallocation of resources that results from taking a local perspective to a larger problem, and to redirect resources into an integrated model of service delivery. OR thus provides another tool for policy makers to analyze the benefits of integrating services at a primary care level.

### 3.3 Example: A simulation model for capacity planning at HIV clinics

**Figure 1:**
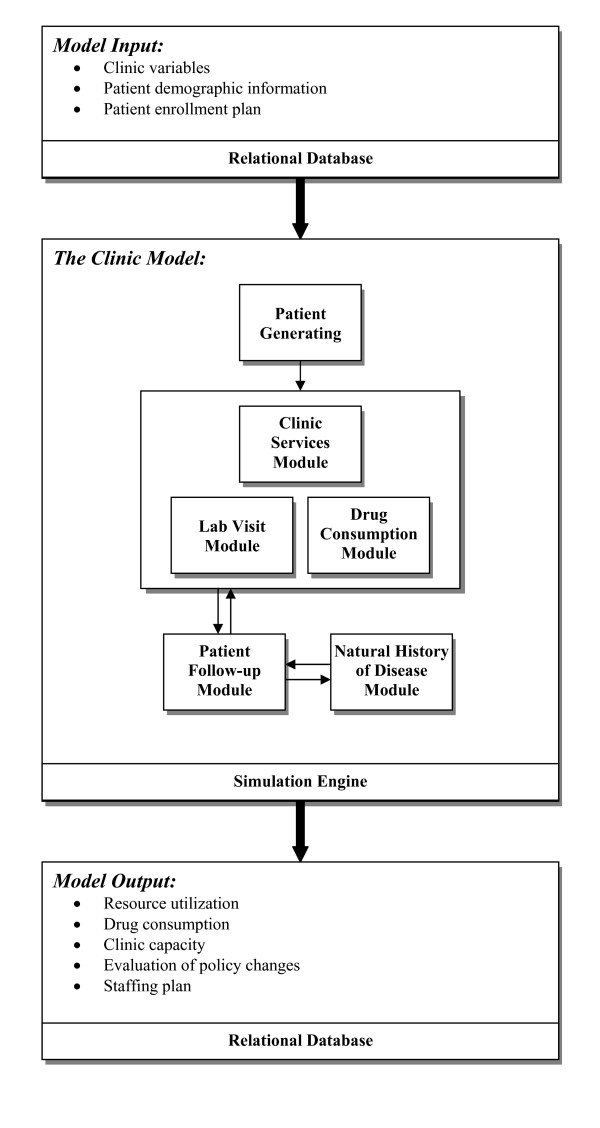
Schematic diagram of simulation model of HIV clinic operations.

In 2005–6, we created a stochastic simulation model to assist capacity planning for HIV treatment clinics, diagramed in Figure 1[37,38]. This simulation model was designed to capture patient characteristics (WHO stage, CD4 count distribution, attrition), disease progression (CD4 decline), treatment protocols, human resource utilization, and the competition for limited resources in a single clinic. We modeled the clinic as a set of interconnected work stations representing different working cadres (e.g., clerk, nurse, doctor). An enrollment plan of the clinic is used as one input to the simulation. Patients scheduled by the enrollment plan, as well as random arrivals, are created by the simulation program and are routed to the clinic, visiting different stations in based on health status (which the model assigns to each patient based on inputted values and evidence-based assumptions) and country-specific treatment protocols (based on health ministry publications). Clinical details ranging from the treatment process, logistics, staffing, to the demand for drugs, are considered in the clinic visit. After each visit, patients may come back or be lost to follow-up visits. During this time, each individual patients disease progression or biological reactions to treatment are modeled. In this way, the simulation program can be used to represent aggregate patient visit dynamics to determine maximal enrollment and visit capacity under steady-state clinic operations in the setting of user-manipulated resource constraints.

In early 2008, we created a simulation model to provide a service benchmarking framework, quantifying the physician time saved in a task-shifting program in Rwanda that gave primary HIV treatment responsibility to nurses working under physician supervision [39]. Using data from this pilot program in three health centers, we were able to estimate the person-hour savings of a critical resource, in this case physicians, by modeling the impact of varying provider assignment, the number and duration of visits, and physician time spent supporting nursing staff. We estimated that if task-shifting were scaled up to the national level it would reduce the demand on public-sector physicians for HIV services by 78%. The analysis suggests that implementation of such a program could allow the government of Rwanda to scale-up HIV treatment without overburdening this component of its existing healthcare system.

By quantifying the outcomes of task-shifting and other programmatic changes in HIV care and treatment, models like these can assist planners in analyzing and optimizing future medical resource allocation. Similar tools can also provide forecasts for key resources, including staffing, space, drug volumes, laboratory kits and equipment to support operational decisions such as the choice of treatment protocols.

### 3.4 Barriers to Adoption of OR Methodologies

The relative absence of OR methodologies from global HIV treatment scale-up activities, especially at the operational level, suggests that barriers exist to their implementation. These are likely to include technical complexity of some of the applications (e.g., involving extensive data analysis and computer-based modeling) and the need for outside content experts to assist local governments and their technical support agencies in the appropriate application of various methodologies to specific problems. More fundamentally, OR-based assessments require investment in the collection, storage, and accessibility of valid, current data to yield accurate and applicable conclusions. Funding of this type of operations-related data collection has not always been prioritized in the rush to scale up "emergency" treatment; whether this was the intention of major funding organizations (rather than simply a consequence of public health planners and practitioners overwhelmed by immediate treatment needs) is under debate [5]. Clearly, development of an OR workforce that is more cognizant of the specific needs and limitations of working in a health setting – especially in resource-limited settings – can only help this situation. Consequently, one concrete action that may speed the application of OR methodologies to HIV scale up will be "cross-training" of engineers in public health and of public health and medical professionals in health systems engineering, either through joint degree programs, special concentrations in existing programs, or post-graduate professional training.

## 4. Discussion

Operations research, the applied field of engineering that is focused on the efficient use of scarce resources, holds great promise to assist in efforts to craft effective and successful scale-up of HIV care and AIDS treatment in resource-limited countries. We have highlighted several areas that parallel HIV scale-up activities and have benefited from OR analyses. However, a number of obstacles to the wide adoption of OR approaches in resource-limited settings, where there are considerable differences in terms of decision making processes when compared to developed countries, remain. A direct transposition of OR techniques and approaches from this setting to another will no doubt encounter many difficulties and may produce misleading results. It is also necessary to take into account the environments in which OR projects are to be carried out. Ethical, cultural, and political considerations, which go beyond maximizing the cost-effectiveness objectives, need to be considered carefully. Furthermore, as noted in a recent review of the PEPFAR program, the general lack of outcomes and operational data – which is being remedied in many programs – is a serious hindrance to carrying out certain OR projects, such as forecasting [5].

In addition to the potential impediments in the field, to date only limited funding has been available for operations-related work in scale-up, and what monies are available for this line of investigation and intervention typically have been underutilized. GFATM, for example, allows up to 10% of each grant to be allocated for operations research, but this provision is rarely used by countries and the research community is rarely represented on Country Coordinating Mechanisms (CCMs) [40]. The recent Sydney Declaration, supporting the allocation of 10% of HIV global programming funding on research related to operations, has highlighted the need for and benefits of OR and related study, echoing the results of the evaluation carried out on the first years of the PEPFAR program [5].

## 5. Conclusion

We stand at a critical moment in the global campaign to scale up treatment for HIV-infected people, one in which the magnitude of the remaining task threatens to overwhelm the hard-fought gains that have brought treatments to millions who recently had none. This paper highlights the potential application of classic operations research approaches to a variety of logistical issues that lie at the heart of the scale-up process. HIV-related operational research has provided ministries of health, non-governmental organizations, public health professionals, and clinic managers with a better understanding of impact and cost-effectiveness of various intervention and treatment programs, as well as quantitative data about clinic capacity, performance and other types of outcomes. The engineering field of OR, with its toolbox of systematic quantitative approaches, can provide additional techniques to understand the data unearthed by engaging in and reporting from clinical operations. We believe that using these analytical techniques will allow clinic managers to get even more utility out of their operational research, and will provide insight into management strategies that can minimize the cost of operations while maximizing clinicians' ability to provide high-quality medical care.

## Competing interests

The authors declare that they have no competing interests.

## Authors' contributions

WX, NH, MEO, and WRR conceived of the concept of the paper. WX and NH wrote the first draft and all authors contributed to that and to subsequent drafts of the paper.

## Pre-publication history

The pre-publication history for this paper can be accessed here:


